# Surgical decision-making in cholesteatoma-induced labyrinthine fistula: hearing outcomes in a tertiary referral center

**DOI:** 10.1007/s00405-026-10243-7

**Published:** 2026-05-05

**Authors:** İlker Akyıldız, Murat Günay, Sibel Alicura Tokgöz, Murad Mutlu, Samet Özlügedik, Muharrem Dağlı

**Affiliations:** Department of Otolaryngology–Head and Neck Surgery, Etlik City Hospital, Ankara, Türkiye

**Keywords:** Cholesteatoma, Labyrinthine fistula, Hearing preservation, Partial labyrinthectomy

## Abstract

**Objective:**

To evaluate surgical outcomes in patients with cholesteatoma-induced labyrinthine fistula, with particular emphasis on hearing preservation across different fistula stages and in selected cases requiring partial labyrinthectomy.

**Methods:**

A retrospective analysis was conducted on patients who underwent surgery for cholesteatoma with intraoperatively confirmed labyrinthine fistula at a tertiary referral center. Demographic data, clinical findings, radiological characteristics, fistula localization, size, and stage were recorded. Fistulae were classified according to the Dornhoffer–Milewski system. Surgical management was individualized according to fistula stage and intraoperative findings. Preoperative and 6-month postoperative bone-conduction (BC) thresholds were compared to assess audiological outcomes. Fistula size was measured on preoperative high-resolution computed tomography, and its relationship with postoperative hearing outcomes was analyzed.

**Results:**

Among 230 patients operated on for cholesteatoma, 39 (17.0%) had a labyrinthine fistula. The lateral semicircular canal was the most frequently involved site (84.6%). According to fistula stage, 17.9% were grade I, 53.8% grade II, and 28.2% grade III. Overall, mean BC thresholds showed no statistically significant change at 6 months postoperatively (*p* = 0.176). Hearing outcomes remained stable in grade I and grade II fistulae, whereas grade III fistulae demonstrated heterogeneous audiological outcomes, with a trend toward postoperative deterioration overall and slight improvement in selected patients managed without partial labyrinthectomy, without reaching statistical significance. Partial labyrinthectomy was performed in five patients with extensive grade III fistulae; although postoperative BC thresholds increased, residual hearing was preserved in all cases and no patient progressed to complete anacusis. Transient postoperative vestibular symptoms resolved with conservative management, and no patient developed persistent disabling vertigo.

**Conclusion:**

Surgical management of cholesteatoma-induced labyrinthine fistula enables effective disease control with acceptable functional outcomes. Hearing preservation is generally achievable in early-stage fistulae, and even in selected advanced cases requiring partial labyrinthectomy, the applied surgical approach may allow preservation of residual hearing. These findings support an individualized, experience-based surgical approach guided by fistula characteristics and intraoperative assessment rather than fistula stage alone.

## Introduction

Cholesteatoma is a destructive pathology of the middle ear that may erode surrounding bony structures and extend into the labyrinth, resulting in significant auditory and vestibular morbidity [1, 2]. Among its potential complications, labyrinthine fistula represents one of the most challenging conditions encountered in otologic surgery, as it directly threatens inner-ear function and complicates surgical decision-making [3, 4]. The reported prevalence of labyrinthine fistula in cholesteatoma surgery varies widely, reflecting differences in disease stage, referral patterns, and diagnostic criteria across institutions [2, 5].

The lateral semicircular canal is the most commonly affected site due to its close anatomical relationship with the epitympanum and its susceptibility to chronic inflammatory erosion [2, 4, 6, 7]. Depending on the extent of bony and membranous labyrinth involvement, fistulae may present with a broad spectrum of clinical manifestations, ranging from subtle vestibular symptoms to overt vertigo and sensorineural hearing loss [2, 3, 8, 9]. However, clinical findings alone are often insufficient to reliably predict the presence or severity of labyrinthine fistula, particularly when the fistula site is partially covered by the cholesteatoma matrix [2–4].

From a surgical perspective, the management of labyrinthine fistula requires a careful balance between complete disease eradication and preservation of inner-ear function [1, 3, 4, 10]. While early-stage fistulae are generally associated with favorable hearing outcomes, advanced disease—especially cases with membranous labyrinth involvement—carries a higher risk of postoperative hearing deterioration [9, 11, 12]. Despite advances in imaging techniques and the introduction of various hearing-preservation strategies, the optimal surgical approach, particularly in cases with extensive labyrinthine involvement, remains a subject of debate [4, 9, 10, 12, 13].

The present study aims to evaluate the clinical characteristics, surgical management, and functional outcomes of patients with cholesteatoma-induced labyrinthine fistula treated at a tertiary referral center. Particular emphasis is placed on audiological outcomes across different fistula stages and on selected cases requiring partial labyrinthectomy. By presenting this experience-based series, we aim to provide practical insight to support individualized surgical decision-making in this complex clinical setting [3, 4, 12].

## Materials and methods

### Study design and ethical considerations

This retrospective study was conducted at the Department of Otolaryngology, Etlik City Hospital, a tertiary referral center. The study protocol was approved by the institutional ethics committee and was performed in accordance with the principles of the Declaration of Helsinki. Owing to the retrospective nature of the study, the requirement for informed consent was waived (Approval No: AEŞH-BADEK1-2026-165).

### Patient selection

**Fig. 1:**
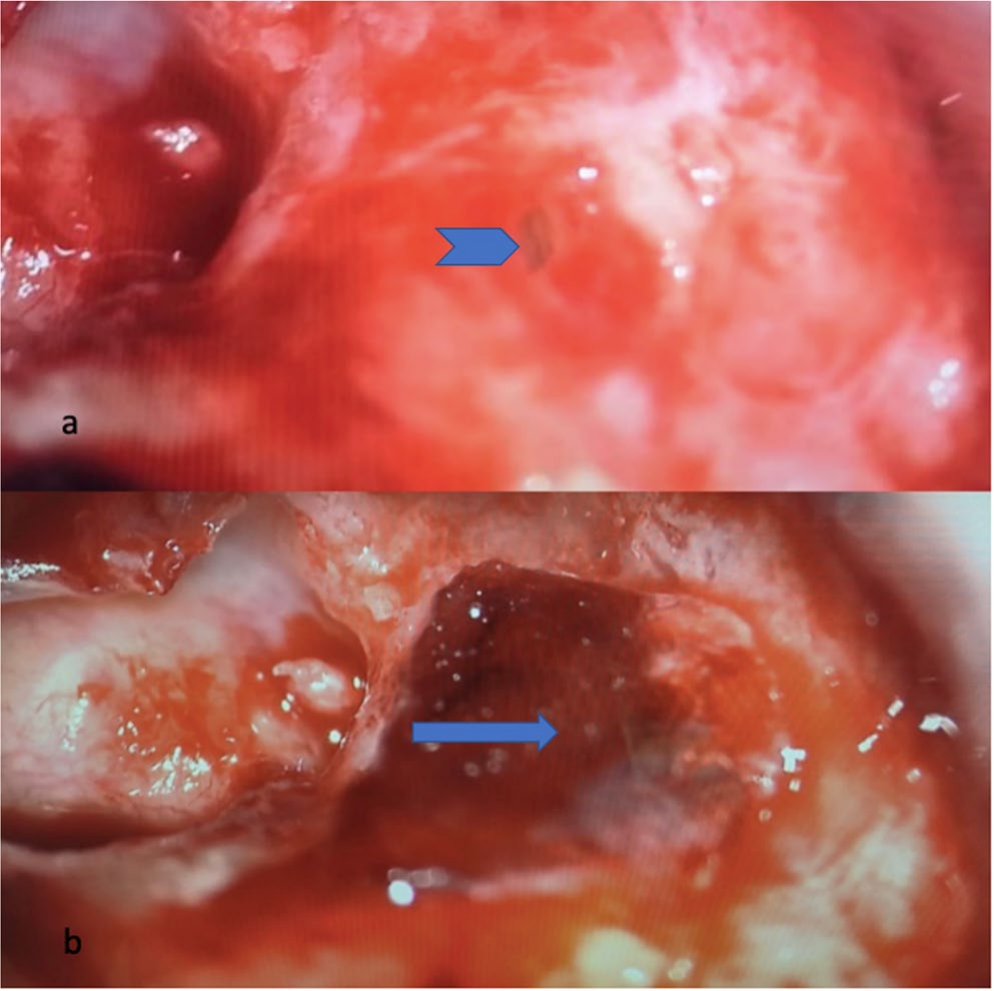
Intraoperative findings and repair of a grade II labyrinthine fistula. (**a**) Grade II labyrinthine fistula characterized by erosion of the bony labyrinth with preservation of the membranous labyrinth (arrowhead). (**b**) Repair of the fistula using temporalis muscle fascia following complete disease clearance (arrow)

**Fig. 2:**
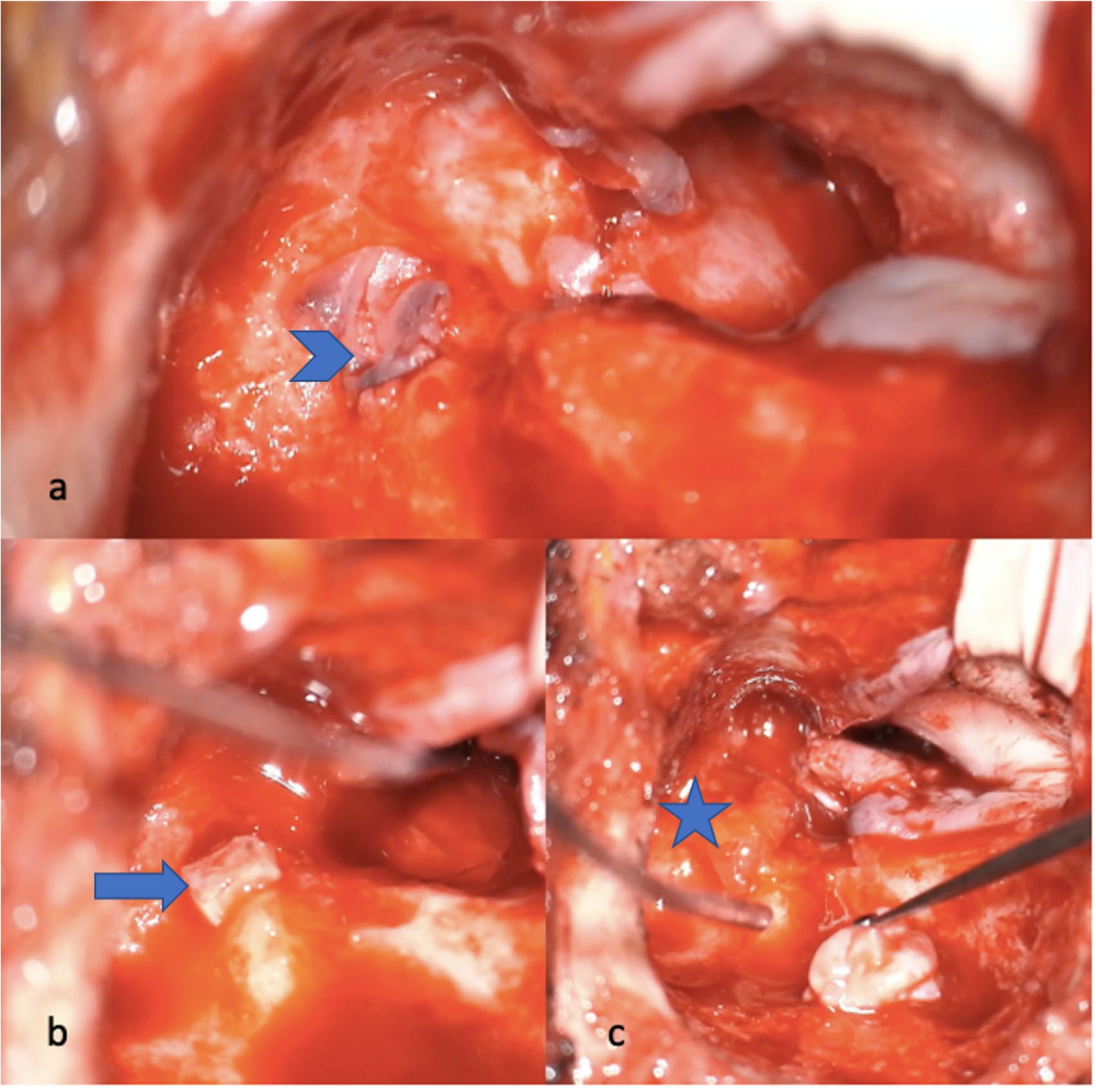
Intraoperative findings and repair of a grade III labyrinthine fistula. **a**. Cholesteatoma eroding and exposing the membranous labyrinth, consistent with a grade III labyrinthine fistula. Removal of the cholesteatoma matrix revealed disruption of the membranous labyrinth, consistent with a grade III fistula (arrowhead). **b**. Reconstruction of the fistula defect using tragal cartilage following complete removal of the cholesteatoma (arrow). **c**. Reinforcement of the repair with temporalis muscle fascia (star)

**Fig. 3:**
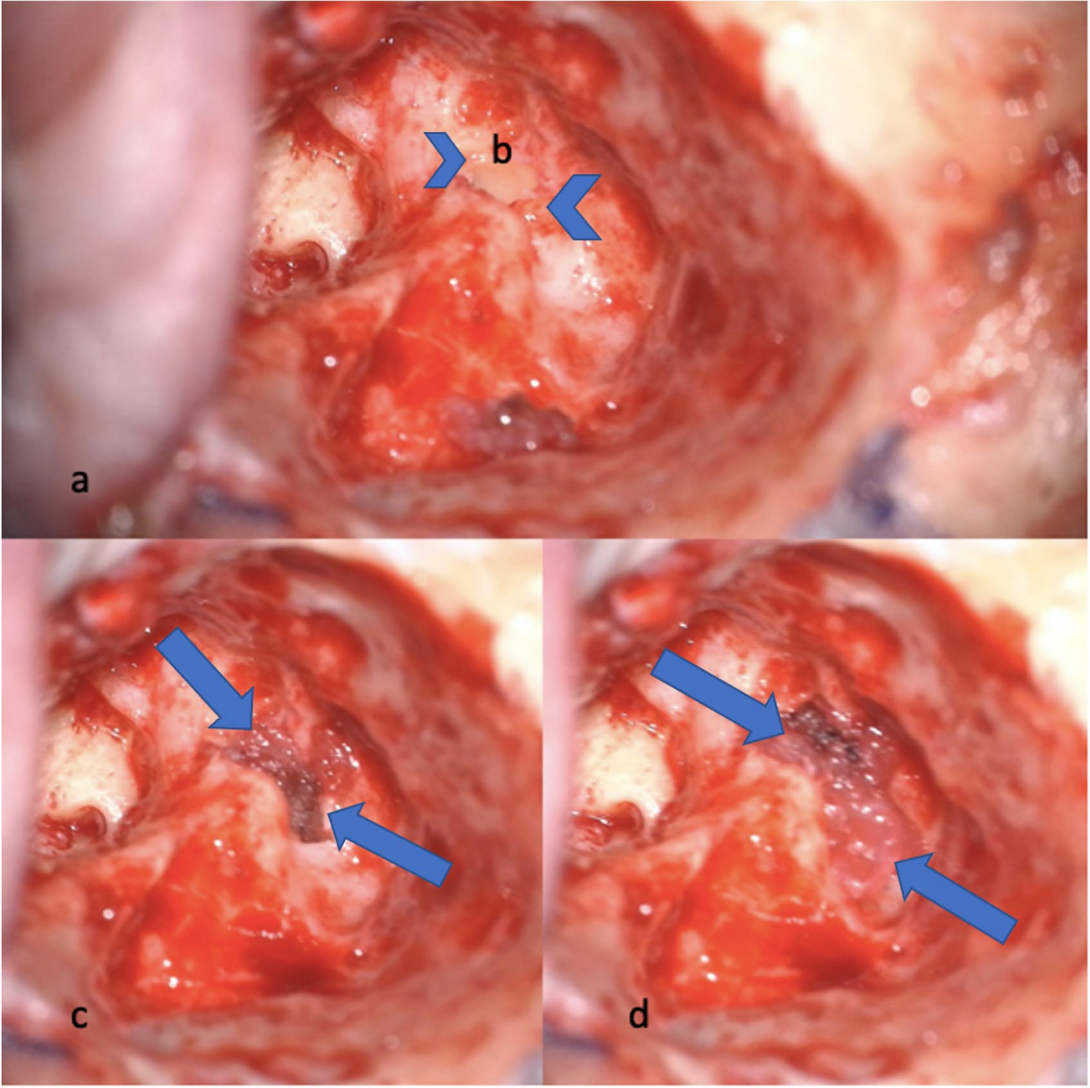
Intraoperative findings and stepwise repair following partial labyrinthectomy in a grade III labyrinthine fistula. **a**. Intraoperative view after complete removal of the cholesteatoma, demonstrating partial labyrinthectomy of the superior semicircular canal (arrowhead). **b**. Intraoperative view of the ampullary end of the superior semicircular canal. (arrowhead). **c**. Repair of the partial labyrinthectomy defect using temporalis muscle (arrow). **d**. Final reconstruction following partial labyrinthectomy using temporalis muscle (arrow)

Medical records of patients who underwent surgery for chronic otitis media with cholesteatoma between September 2022 and November 2025 were reviewed. Patients were included if they had a diagnosis of cholesteatoma confirmed by clinical and radiological evaluation and if a labyrinthine fistula was identified intraoperatively and/or on high-resolution computed tomography (HRCT). Only patients with complete clinical records and available preoperative and postoperative audiometric data were included in the analysis. Patients without cholesteatoma, those without evidence of labyrinthine fistula, or those with incomplete documentation were excluded (Figs. 1, 2, and 3).

### Preoperative evaluation

All patients underwent a standardized preoperative assessment, including otoendoscopic examination, fistula testing, pure-tone audiometry, HRCT of the temporal bone, and magnetic resonance imaging with diffusion-weighted sequences when indicated.

Audiological evaluation focused primarily on bone-conduction thresholds. Labyrinthine fistulae were classified according to the Dornhoffer–Milewski grading system as follows: grade I, bony erosion without involvement of the membranous labyrinth; grade II, exposure of the perilymphatic space with an intact membranous labyrinth; and grade III, disruption of the membranous labyrinth or an open perilymphatic fistula [3, 10].

### Radiological assessment

Fistula size was routinely measured in millimeters on preoperative high-resolution computed tomography images [13]. In patients with multiple fistula sites, the largest measured defect was used for analysis. The relationship between fistula size and postoperative changes in bone-conduction thresholds was evaluated using Spearman correlation analysis across all fistula stages. Radiological findings were reviewed in conjunction with intraoperative observations to ensure accurate characterization of fistula localization, extent, and surrounding bony erosion.

All patients underwent diffusion-weighted MRI at 6 months as part of the postoperative follow-up protocol.

### Surgical technique

All surgical procedures were performed under general anesthesia by experienced otologic surgeons using a consistent surgical philosophy aimed at complete disease eradication with preservation of inner-ear function whenever feasible [1, 3, 4]. In the present series, all patients were managed with a canal wall down (CWD) technique due to the presence of advanced, extensive, and often long-standing untreated cholesteatoma. Given the complexity of these cases in a tertiary referral setting, this approach was preferred to ensure complete disease eradication and minimize the risk of residual or recurrent disease.

The cholesteatoma matrix was carefully removed, and dissection over the fistula site was performed with particular attention to minimizing labyrinthine trauma. Fistula repair was tailored according to fistula stage and intraoperative findings. Grade I and II fistulae were repaired using temporalis muscle fascia, whereas grade III fistulae were reinforced with a combination of tragal cartilage and temporalis fascia [1, 7, 9].

Partial labyrinthectomy was performed in selected cases with advanced grade III fistula when the cholesteatoma matrix could not be safely dissected from the membranous labyrinth, particularly in the presence of extensive endosteal erosion or suspected membranous labyrinth involvement. In these cases, complete disease removal was prioritized over matrix preservation to minimize the risk of residual disease. In these cases, the defect was obliterated using temporalis muscle and abdominal fat to achieve secure sealing and to minimize perilymph leakage [4, 10].

### Postoperative management and follow-up

Postoperatively, patients received standard medical therapy, including intravenous antibiotics and systemic corticosteroids. Vestibular suppressants were administered when necessary.

Audiological evaluation was repeated at 6 months postoperatively to assess changes in bone-conduction thresholds. Patients were followed clinically to monitor vestibular symptoms, wound healing, and postoperative complications.

### Statistical analysis

Statistical analyses were performed using SPSS software (IBM Corp., Armonk, NY, USA). Preoperative and postoperative bone-conduction thresholds were compared using the Wilcoxon signed-rank test. Subgroup analyses were conducted using the Mann–Whitney U test and the Kruskal–Wallis test, as appropriate. Correlations between continuous variables were evaluated using Spearman correlation analysis. A p value of less than 0.05 was considered statistically significant.

## Results

### Patient characteristics

Between September 2022 and November 2025, a total of 230 patients underwent surgery for cholesteatoma at our institution. Among these, 39 patients (17.0%) were found to have an intraoperatively confirmed labyrinthine fistula and constituted the study cohort. The mean age of the patients was 50.1 ± 17.3 years (range, 16–82 years). The cohort included 25 males (64.1%) and 14 females (35.9%) (Table 1). Preoperative vertigo was reported by 24 patients (61.5%), while the fistula test was positive in 18 patients (46.2%).

**Table 1:** Demographic and clinical characteristics of the study cohort

Characteristic	Result
Number of patients (n)	39
Age, years (mean ± SD)	50.1 ± 17.3
Age range, years	16–82
Sex, n (%)	
Male	25 (64.1%)
Female	14 (35.9%)

### Fistula localization, size, and stage

The lateral semicircular canal was the most frequently involved site, with isolated involvement observed in 33 patients (84.6%). Isolated superior semicircular canal fistulae were identified in 3 patients (7.7%), while combined lateral and superior canal involvement was observed in 3 patients (7.7%). According to the Dornhoffer–Milewski classification, 7 fistulae (17.9%) were classified as grade I, 21 (53.8%) as grade II, and 11 (28.2%) as grade III. Fistula size, measured on preoperative HRCT, varied across patients and was generally larger in advanced-stage disease (Table 2).

**Table 2:** Anatomical characteristics of labyrinthine fistulae

Characteristic	Result
Involved semicircular canal, n (%)	
Isolated lateral semicircular canal	33 (84.6%)
Isolated superior semicircular canal	3 (7.7%)
Combined lateral and superior canal	3 (7.7%)

### Surgical management

All patients underwent complete cholesteatoma removal with fistula repair tailored to fistula stage and intraoperative findings. Temporalis muscle fascia was used for the repair of grade I and grade II fistulae, whereas combined tragal cartilage and temporalis fascia reinforcement was applied in grade III cases (Table 3). Partial labyrinthectomy was performed in five patients with extensive grade III fistulae, followed by obliteration of the defect with temporalis muscle and abdominal fat. The interval between the last preoperative HRCT scan and surgery varied among patients but did not influence intraoperative decision-making.

**Table 3:** Surgical management and repair techniques according to fistula grade

Fistula grade	Number of patients (*n*)	Repair /Reconstructiontechnique
Grade I	7 (17.9%)	Temporalis fascia
Grade II	21 (53.8%)	Temporalis fascia
Grade III (without partial labyrinthectomy)	6 (15.3%)	Tragal cartilage+ temporalis fascia
Grade III (with partial labyrinthectomy)	5 (12.8)	Temporalis muscle + abdominal fat obliteration

### Audiological outcomes

For the entire cohort, mean preoperative bone-conduction (BC) thresholds were 35.3 ± 21.9 dB, compared with 37.9 ± 21.5 dB at the 6-month postoperative evaluation, with no statistically significant difference (*p* = 0.176) (Table 4).

**Table 4:** Audiological outcomes according to fistula grade and surgery

Group	*n*	Preoperative BC (dB, mean ± SD)	Postoperative BC (dB, mean ± SD)	*p*-value
Entire cohort	39	35.3 ± 21.9	37.9 ± 21.5	0.176
Grade I	7	24.7 ± 13.0	25.6 ± 17.7	0.75
Grade II	21	40.8 ± 21.5	41.6 ± 20.8	0.24
Grade III (without partial labyrinthectomy)	6	43.0 ± 28.3	38.3 ± 27.3	0.28
Grade III (with partial labyrinthectomy)	5	18.8 ± 6.8	31.4 ± 8.7	0.063

Stage-based analysis demonstrated generally stable BC thresholds in patients with grade I and grade II fistulae. In grade I cases (*n* = 7), mean BC thresholds changed from 24.7 ± 13.0 dB preoperatively to 25.6 ± 17.7 dB postoperatively (*p* = 0.75). Similarly, in grade II cases (*n* = 21), values changed from 40.8 ± 21.5 dB to 41.6 ± 20.8 dB (*p* = 0.24).

In patients with grade III fistulae, audiological outcomes differed according to surgical approach. Among patients managed without partial labyrinthectomy (*n* = 6), mean BC thresholds decreased from 43.0 ± 28.3 dB to 38.3 ± 27.3 dB postoperatively, although this change did not reach statistical significance (*p* = 0.28). In contrast, patients who underwent partial labyrinthectomy (*n* = 5) demonstrated greater postoperative deterioration, with mean BC thresholds increasing from 18.8 ± 6.8 dB to 31.4 ± 8.7 dB, approaching statistical significance (*p* = 0.063). Despite this deterioration in the partial labyrinthectomy subgroup, residual hearing was preserved in all patients, and no case progressed to complete anacusis.

In patients with longer follow-up, no clinically significant changes in hearing thresholds were observed beyond the 6-month postoperative evaluation, supporting the use of 6-month outcomes as representative of stable hearing levels.

### Fistula size and audiological outcomes

No statistically significant correlation was identified between fistula diameter and postoperative changes in bone-conduction thresholds across fistula stages.

In grade I fistulae (*n* = 7), a moderate negative trend was observed between fistula size and hearing change, although this association did not reach statistical significance (Spearman *r* = − 0.54, *p* = 0.22). In grade II cases (*n* = 21), no significant correlation was identified between fistula size and postoperative BC changes (*r* = − 0.32, *p* = 0.16). In patients with grade III fistulae managed without partial labyrinthectomy (*n* = 6), size–outcome analysis demonstrated a moderate positive trend that did not reach statistical significance (*r* = 0.60, *p* = 0.21). Similarly, in grade III patients who underwent partial labyrinthectomy (*n* = 5), no significant association was observed between fistula size and postoperative hearing deterioration (*r* = 0.38, *p* = 0.53).

Overall, these findings indicate that fistula size alone is insufficient to reliably predict postoperative auditory outcome and that functional results are likely influenced by multiple factors, including disease extent, membranous labyrinth integrity, and intraoperative surgical technique.

### Vestibular outcomes and follow-up

Transient postoperative vestibular symptoms were observed in some patients, particularly those with advanced-stage fistulae or those who underwent partial labyrinthectomy. These symptoms resolved with conservative management during follow-up. No patient developed persistent or disabling vertigo requiring further intervention.

The mean follow-up duration was 16.8 ± 8.7 months. During the follow-up period, no evidence of intralabyrinthine recurrence was detected on diffusion-weighted MRI, except in one patient in whom a small (2 mm) cholesteatoma pearl was identified within the mastoid cavity.

## Discussion

The management of cholesteatoma-induced labyrinthine fistula remains one of the most challenging aspects of otologic surgery, as it requires a careful balance between effective disease clearance and preservation of inner-ear function [3, 4, 6, 7, 9, 10]. Previous systematic reviews and meta-analyses have reported generally favorable hearing outcomes in early-stage fistulae, whereas advanced disease is associated with a higher risk of postoperative deterioration [12]. Our findings are consistent with this evidence, demonstrating stable bone-conduction thresholds in grade I and grade II fistulae and a tendency toward greater deterioration in grade III cases [11, 14]. However, the absence of a statistically significant hearing decline across fistula stages may suggest a limited predictive value of fistula grade alone; however, this observation should be interpreted with caution, particularly in light of the limited number of patients with stage III disease.

The prevalence of labyrinthine fistula in the present series was relatively high compared with most published reports [1, 5]. This finding is likely attributable to the characteristics of our study population, as our institution serves as a tertiary referral center managing a high proportion of advanced and previously untreated cholesteatoma cases. Similar observations have been reported in large referral-based series, in which the likelihood of labyrinthine fistula increases with disease extent and duration [7, 8, 13]. Therefore, the fistula rate observed in our cohort should be interpreted as a reflection of case complexity and referral bias rather than an epidemiological deviation.

Preoperative vestibular symptoms were frequently observed in the present cohort, whereas the fistula test demonstrated limited sensitivity [2, 3, 9]. This discrepancy has been well documented in previous studies and is generally attributed to the presence of an intact or partially intact cholesteatoma matrix covering the fistula site, which may attenuate pressure transmission to the labyrinth [2, 4]. Consequently, both vestibular symptoms and fistula test results may underestimate the true extent of labyrinthine involvement. Our findings further indicate that neither vertigo nor a positive fistula test represents a reliable standalone marker of fistula severity, underscoring the importance of correlating clinical findings with radiological imaging and intraoperative assessment [8, 15].

Consistent with previous reports, the lateral semicircular canal was the most frequently involved site in our series [6, 7, 11]. This predilection is attributed to its close anatomical relationship with the epitympanum and its susceptibility to chronic inflammatory erosion. In contrast, superior semicircular canal involvement was uncommon and occurred either in isolation or in combination with lateral canal fistulae [5]. In such cases, particular caution is required because of the proximity to the labyrinthine segment of the facial nerve, which increases the risk of iatrogenic injury during surgical dissection [15]. Overall, the anatomical distribution observed in our cohort aligns with previously reported patterns and supports the concept that fistula localization reflects disease extent rather than a distinct clinical entity.

Fistula size and stage have traditionally been considered important determinants of postoperative auditory outcome; however, their relationship with hearing preservation remains complex [3, 10, 11, 13]. In our series, early-stage fistulae were generally associated with stable postoperative bone-conduction thresholds, whereas advanced-stage fistulae showed a tendency toward greater deterioration [14]. Nevertheless, this change did not reach statistical significance, indicating that fistula stage alone is insufficient to reliably predict functional outcome. Previous studies have similarly suggested that additional factors—including membranous labyrinth integrity, intraoperative manipulation of the fistula site, and the extent of surrounding inflammation—play critical roles in hearing preservation [10, 13]. Accordingly, fistula size and stage should be interpreted within a broader intraoperative context rather than as isolated predictors of postoperative hearing outcome.

The optimal surgical management of labyrinthine fistula remains debated, particularly with regard to the extent of matrix removal and the choice of repair technique [4, 9, 10, 15]. The optimal management of the cholesteatoma matrix overlying a labyrinthine fistula remains controversial. Some authors advocate complete removal of the matrix to ensure total disease eradication and reduce the risk of residual or recurrent cholesteatoma. However, this approach carries a potential risk of iatrogenic injury to the membranous labyrinth, which may result in irreversible sensorineural hearing loss or vertigo. Alternatively, matrix preservation has been proposed as a more conservative strategy aimed at protecting inner-ear function, particularly in cases with an intact membranous labyrinth. While this technique may reduce the risk of immediate cochlear damage, it may also increase the likelihood of residual disease and necessitate closer follow-up or staged surgery. In our practice, the decision was individualized based on intraoperative findings, balancing the goal of complete disease eradication against the risk of inner-ear injury.

In the present series, a consistent surgical strategy was adopted, prioritizing complete disease clearance while tailoring fistula repair according to fistula stage and intraoperative findings. Temporalis fascia was used for grade I and II fistulae, whereas combined cartilage and fascia reinforcement was preferred in grade III cases to provide greater structural support in advanced disease [11, 14]. Although various hearing-preservation techniques, including underwater dissection, have been proposed, their applicability remains limited in extensive disease requiring more direct surgical intervention [9, 15]. Our findings suggest that successful fistula management depends primarily on careful intraoperative judgment, appropriate reconstruction, and individualized adaptation of surgical strategy.

The relationship between fistula size and postoperative auditory outcome remains controversial in the literature [12, 13]. In the present study, no statistically significant correlation was identified between fistula diameter and postoperative changes in bone-conduction thresholds across different fistula stages. Although moderate trends were observed in certain subgroups, these associations did not reach statistical significance, suggesting that fistula diameter alone is insufficient to reliably predict functional outcome. These findings support the concept that meticulous disease eradication and secure fistula sealing may mitigate the potential adverse effects of larger defects on cochlear function [10, 13].

Of particular interest, patients with grade III fistulae managed without partial labyrinthectomy demonstrated a tendency toward postoperative improvement in bone-conduction thresholds. Although not statistically significant, this observation suggests that careful matrix removal and effective fistula reconstruction may exert a protective effect on cochlear function in selected cases [10, 13]. In contrast, patients who underwent partial labyrinthectomy demonstrated measurable postoperative deterioration, consistent with previous reports [6, 12]. Nevertheless, residual hearing was preserved in all cases, and no patient progressed to complete anacusis, in line with earlier studies emphasizing the importance of careful reconstruction in limiting irreversible inner-ear damage [8]. However, the number of patients undergoing partial labyrinthectomy in our series was limited, and therefore these findings should be interpreted with caution. Given the rarity of such advanced cases, the present results should be considered exploratory and hypothesis-generating rather than definitive.

Postoperative vestibular symptoms in the present cohort were generally transient and self-limited [2, 4]. Early postoperative dizziness was observed in some patients, particularly those with advanced fistulae or extended surgical procedures, but symptoms resolved with conservative management. Notably, no patient developed persistent or disabling vertigo requiring additional intervention. This finding underscores the importance of careful labyrinthine handling and appropriate fistula reconstruction in minimizing long-term vestibular morbidity.

All patients in the present series underwent diffusion-weighted MRI at 6 months as part of a standardized follow-up protocol. No cases of intralabyrinthine recurrence were identified. The overall recurrence rate was low, likely reflecting the use of a canal wall down technique in all patients. A single case of limited recurrent cholesteatoma pearl, measuring approximately 2 mm, was detected within the mastoid cavity rather than the labyrinth in a patient who had undergone partial labyrinthectomy. This lesion was considered clinically limited and is planned for endoscopic removal.

Several limitations of this study should be acknowledged. Its retrospective design and single-center setting may limit the generalizability of the findings. In addition, the relatively small number of patients undergoing partial labyrinthectomy restricts the statistical power of subgroup analyses. Audiological evaluation was primarily based on bone-conduction thresholds, and more comprehensive functional measures, such as speech perception outcomes, were not consistently available. Nevertheless, the homogeneity of surgical strategy and standardized follow-up provide a valuable perspective on real-world management of cholesteatoma-induced labyrinthine fistula.

Taken together, the present findings support an individualized approach to the surgical management of labyrinthine fistula [4, 6, 12, 15]. Rather than relying on fistula stage alone, surgical strategy should be guided by disease extent, radiological findings, and real-time intraoperative assessment. Such an experience-based and adaptable approach may facilitate optimal disease control while preserving functional outcomes.

## Conclusion

Cholesteatoma-induced labyrinthine fistula represents a complex surgical entity requiring a careful balance between complete disease eradication and preservation of inner-ear function. In this series, stable audiological outcomes were generally observed in early-stage fistulae, whereas advanced disease was associated with a higher risk of postoperative hearing deterioration. Notably, even in selected cases requiring partial labyrinthectomy, residual hearing may be preserved in selected cases, and no patient progressed to complete anacusis. These findings emphasize that fistula management should be individualized and guided by fistula characteristics and intraoperative assessment rather than fistula stage alone. An experience-based and adaptable surgical strategy may facilitate effective disease control while maintaining acceptable functional outcomes, even in advanced cases. 
